# A cross sectional study: the knowledge, attitude, perception, misconception and views (KAPMV) of adult family members of people living with human immune virus-HIV acquired immune deficiency syndrome-AIDS (PLWHA)

**DOI:** 10.1186/s40064-015-1541-2

**Published:** 2015-12-12

**Authors:** Akshaya Srikanth Bhagavathula, Deepak Kumar Bandari, Asim Ahmad Elnour, Akram Ahmad, Muhammad Umair Khan, Mohamed Baraka, Farah Hamad, Abdulla Shehab

**Affiliations:** Department of Clinical Pharmacy, University of Gondar College of Medicine and Health Sciences, Gondar, Ethiopia; Department of Clinical Pharmacy, Vagdevi College of Pharmacy, Warangal, Telangana India; Pharmacology Department, College of Medicine and Health Sciences, UAE University, Dubai, UAE; Department of Clinical Pharmacy, UCSI University, Cheras, Kuala Lumpur, Malaysia; Department of Clinical Pharmacy, College of Clinical Pharmacy, University of Dammam Eastern Province, Dammam, Kingdom of Saudi Arabia; Department of Pharmaceutics, College of Pharmacy, Ajman University of Sciences and Technology, Ajman, UAE; Internal Medicine Department, College of Medicine and Health Sciences (CMHS), UAE University, Al Ain, UAE

**Keywords:** People living with human immune virus (HIV) acquired immune deficiency syndrome (AIDS)—PLWHA, PLWHA adult family member, AIDS, HIV, Knowledge attitude perception misconception views (KAP-MV)

## Abstract

We intended to assess knowledge, attitude, perception, misconception and views (KAP-MV) of family members of PLWHA. A cross-sectional retrospective study conducted in Anti-retroviral centre of Mahatma Gandhi Memorial—MGM hospital, Warangal, Telangana, South-India from July to September 2014. A questionnaire containing 41 items was distributed among adult family members accompanying patients living with HIV/AIDS-PLWHA. Level of KAP-MV was categorized into poor (0–28), average (29–55) and good (56–82). Analysis was performed by Pearson’s Chi square, analysis of variance and Spearman’s correlation test on 41 variables using SPSS version 21 and *p* < 0.01. 538 questionnaires were distributed, response rate was (96 %). On knowledge scale, respondents had a mean score of 8.0 ± 1.7, attitude 5.8 ± 3.4, perception 23.4 ± 4.1, misconceptions 8.0 ± 2.1 and views 8.0 ± 3.9. The respondents mean score was 53.2 ± 9.1 (64.9 %). Overall, level of education, marital status, religious beliefs, and employment status has significant (p < 0.001) associations with KAP-MV. Knowledge was significantly correlated with respondents’ attitude (r = −0.15, p < 0.001), perception (0.39; p < 0.001), and views (0.381; p < 0.001). Family members of PLWHA with less knowledge score had more negative attitude, perception and views. Level of education, marital status, religious beliefs and employment status were identified as key barriers. Interventions targeting family members of PLWHA are warranted. Practice implications are as follows:

- Encourage role of family members.
- Deploy interventions.
- Minimize barriers.
- Change misconceptions.

## Background

Verging on forth decade of acquired immune deficiency syndrome-AIDS epidemics, India ranks the third largest human immune virus (HIV) epidemics in the world (National AIDS Control Organization, Department of AIDS Control 2012). In line with National Control Organization (NACO) for AIDS the burden of HIV infected people in India was estimated to be 2.1 million in 2013, (National AIDS Control Organization, Department of AIDS Control 2012). Among the highest prevalence states, Andhra Pradesh accounts for 0.90 % of HIV prevalence followed by Karnataka (0.63 %) and accounts for 53 % of all the HIV/AIDS infected people from south India, (Dandona et al. 2013). Warangal (now in Telangana state) was recorded as the most HIV/AIDS prevalent district in Andhra Pradesh, (Joshua et al. 2011).

Family members of people living with human immune virus-AIDS (PLWHA) directly or indirectly face embarrassment, shame, ostracism and stigma from the community, (Vlassoff et al. 2012). Furthermore, these situations are complicated especially in socio-cultural and traditional community, (Mawar et al. 2005). A study reported that PLWHA individuals and their families were socially isolated and were blamed by the communities in India, (Pallikadavath et al. 2005). These factors impede timely helpseeking with strong bonds between PLWHA and their families to care and support. In addition, the existing evidence of 28 studies (18 of which in Sub-Saharan Africa) as reported in a literature review supports the role of the family members as key predictors in providing care and support for the well-being of PLWHA (Liyun and Li 2013).

Adult family members are the solo responsible individuals caring for PLWHA apart from health professionals. Adult family members are more likely to become the principle caregivers for PLWHA at homes. In addition, emphasis for well-being of PLWHA demonstrated by growing evidence related to the outcomes of family caregivers.

Knowledge, attitude, and practice towards HIV/AIDS vary and have widespread misconceptions including fear of infection transmission, social stigma and personal fear within the family members, (Feng et al. 2009). This is even more so for India, where the impact of HIV/AIDS radiates from an infected person to the whole family. Due to existing discrimination in the society against PLWHA, knowledge, attitude, perception, misconception and views of the family members is less often studied than other individuals. India’s National Family Health Survey-3 (NFHS-3) 2007, reported only 17 % of the women and 33 % of the men had a comprehensive knowledge about HIV prevention and transmission, (International Institute for Population Sciences 2005).

Nevertheless, NFHS-3 data included sample from the general population from rural and tribal areas of India. However, some studies focus on the knowledge, attitudes, and practice of the HIV/AIDS in the general population, health professionals and students, (Meera et al. 2013; Kermode et al. 2005; Shankar et al. 2011). While other studies focus on gendered dimension, stigma, care givers burden and stress, (Bharat et al. 2014; Jeyaseelan et al. 2013; Krishna et al. 2005). However, these studies have been limited to assessing integrated knowledge, attitudes, perception, misconception and views (KAP-MV) of adult family members of PLWHA. Few studies have examined potential differences in knowledge, attitude, risk perception, misconception and adult family member views towards HIV/AIDS and PLWHA.

The evaluation of PLWHA adult family member’s KAP-MV is crucial to tailor the home care, prevention measures to halt transmission and effective interventions for PLWHA. Therefore, having accurate knowledge about HIV/AIDS is important to reduce misconceptions, and to create a more humanitarian attitude and compassionate response of PLWHA families towards HIV/AIDS. To our knowledge, the current study is the first of its kind to make an indepth assessment of integrated KAP-MV of adult family members towards PLWHA in high prevalent Warangal district, Telangana state of South-Central India.

### Ethical approval

The study protocol was approved by Institutional ethical committee of the Mahatma Gandhi Memorial (MGM) institute of Medical Sciences, Warangal, Telangana state.

### Aim

We intended to assess the knowledge, attitude, perception, misconception and views (KAP-MV) of adult family members of PLWHA in South India. The study aim was to delineate the predictors that affect adult family member’s KAP-MV.

### Objectives

We have objectively assessed the KAP-MV of adult family members within a cohort of HIV/AIDS positive patients. The assessment of general knowledge has identified the level of understanding of adult family members towards HIV/AIDS, attitudes have addressed their feelings, perceptions have addressed the possible causes of HIV/AIDS, misconception have investigated their ideas towards certain myth about PLWHA and views examined certain daily contact activity of measures to prevent HIV transmissions.

## Methods

### Study design and setting

A cross-sectional survey was conducted using self-administered questionnaire ("Appendix") among adult family members accompanying PLWHA in anti-retroviral centre of MGM hospital, Warangal-Telangana, South-India. This hospital is 1200 bedded multidisciplinary tertiary care government teaching hospital. Warangal district had a population of 3.58 million in the 2011 census, (Registrar General of India 2011).

### Study population

The population was composed of all adult visitors accompanying patients with HIV/AIDS visiting the main hospital. PLWHA adult family members were informed about the study prior to their participation. Those who agreed to participate were consented.

### Sample size and sample selection

An online sample size calculator “Creative research systems” (Accessed June 2014), was used to determine the number of participants for the survey, by considering 95 % confidence level, with a accuracy of 50 % for the population size of 135,000 antiretroviral therapy (ART) registered patients given a confidential interval of 4.2, the recommended sample size is 542. A systematic random sampling technique was used to select PLWHA family members that includes spouse, parents, siblings, or any of blood relations, presenting with registered ART patients attending ART specialized regional center on each day of data collection. The average attendance at the ART regional center on each month in the last 6 months served as a sample frame of 2197 and by dividing this population by the sample size of 542 in cohort, one out of every four PLWHA attending with family members were selected. One from each adult PLWHA family members were selected to align with the sample representation and obtain diverse opinion from a wide range from them. Further, the research assistants has cross-checked each new client to ensure that no PLWHA family members were selected twice.

### Inclusion and exclusion criteria

The inclusion criteria included been an adult family member of PLWHA, aged more than 18 years, both gender and willing to provide written consent. The healthcare providers, social caregivers, family member children’s (less than 18 years), not willing to participate and not able to provide written consent were all excluded from the study.

### Study survey instrument (KAP-MV)

We have developed structured study survey-questionnaire which was self-administered to collect socio-demographics and the five domains outcome measures (KAP-MV). The questionnaire items used in this study was based on the World Health Organization (WHO) AIDS program knowledge, attitudes, beliefs and practices (KABP) survey in 1990, (World Health Organization 1990).

### KAP-MV survey contents

The questionnaire comprises of 50 closed questions with a dichotomous responses (Yes/No). It was divided into six parts. Part A related to respondent’s socio-demographic background (9 items), Part B was relevant to knowledge regarding HIV/AIDS (5 statements), Part C on risk perception towards possible causes of HIV/AIDS (12 statements), Part D on AIDS attitude scale (5 statements), Part E on respondents views about measure to prevent HIV transmissions (12 statements) and Part F on misconceptions towards HIV/AIDS (5 statements).

### KAP-MV survey validation and reliability

All the questions were translated from English to Telugu (back and forward method) by expert faculty members (number of 6). The Telugu version was used for the study participants. The questionnaire was tested for reliability, psychometric and internal validity. The internal consistency estimate of reliability of test scores (Cronbach’s Alfa) was found to be 0.76 indicating a good construct of KAP-MV. Furthermore, KAP-MV was piloted and post piloting phase the questionnaire was modified to meet the compatibility of local settings and suit the study participants. The results of the piloting phase were not included herein. The questionnaires were completed in the waiting room and took an average of 30 min to complete. The completed questionnaires were collected and retrieved by one investigator [*DKB*] on daily bases from July to September 2014.

### KAP-MV survey outlines

#### Knowledge domain

This domain asked adult family members their basic knowledge about HIV/AIDS as to the meaning of AIDS abbreviation, whether AIDS is a transmittable, hereditary, cured or amendable to vaccine.

#### Attitudes domain: address feelings

The attitudes domain inquire about the feelings of adult family members towards been comfortable talking with PLWHA, feel comfortable working with them, living with them, feel empathy towards AIDS patients and PLWHA deserve free treatment.

#### Perception domain: towards causes of HIV/AIDS

We cited 14 items to address the perception of adult family members of PLWHA towards the causes of HIV/AIDS. The perception of participants towards causes of HIV/AIDS, ("Appendix").

#### Misconception domain: myths about HIV/AIDS

The KAP-MV has examined the misconception of adult family members of PLWHA about their respective infective beloved patients. The following were the addressed statements of misconception: love is a reason for HIV/AIDS, AIDS is a punishment from GOD, AIDS can be treated by holy water, AIDS do not come after marriage and AIDS can be transmitted by a cough.

#### Views domain: about measures to prevent HIV/AIDS transmission

We have addressed the views of adult family members of PLWHA about measures to prevent HIV/AIDS transmission. This has included the followings: avoiding sharing needles and syringes, having sex with only one faithful, uninfected partner, using condoms during sexual intercourse, treating sexual transmitted diseases (STDs) promptly, screening donated blood before transfusion, not sharing toilets with an infected person, not sharing food with an infected person, isolating people living with HIV/AIDS, do not stay with infected person on same house, do not have casual contact with infected person and avoid mosquito bites for HIV transmission.

#### KAP-MV scoring

The scoring mechanism of each section was developed by the researchers. For all the 41 statements (excluding socio-demographics), each correct answer was given a score of 2 while each wrong answer was given a score of 0. Therefore, a respondent could score a maximum of 82 and a minimum of 0 in all sections of questionnaire. Hence, the total score of KAP-MV has ranged from 0 to 82. The higher the score, the better were the respondent’s HIV/AIDS related KAP-MV and less misconception. The overall scores were also categorized into poor (0–28), average (29–55) and good (56–82).

#### KAP-MV procedure

The questionnaire was administered while the adult family members of PLWHA were in the waiting room after explaining nature and anonymity of the survey, and assuring confidentiality of the personal responses. The survey was conducted during the regular ART centre working hours. Participants were provided with pens and were asked to sit apart and were asked not to communicate with each others during administration of the questionnaire so as to encourage spontaneous and honest responses.

### Statistical analysis

The statistical analysis were carried out by SPSS (version 21), which includes frequency count and percentages for socio-demographic variables, Pearson’s Chi square, analysis of variance (one-way ANOVA) and Spearman’s rank order correlation (rho) test was conducted on 41 variables towards KAP-MV reported by families of PLWHA. A *p* value of <0.01 was considered statistically significant.

## Results

The ART regimen was constituted of combination of Nucleoside analogue reverse transcriptase inhibitors (NRTIs) Zidovudine, Lamivudine and Stavuidine (three types) and nonnucleoside analogue reverse transcriptase inhibitors (NNRTIs) Nevirapine and Efavarinz (two types) as ART medications.

### Respondents’ socio-demographic profile

Among 536 respondents, 515 (96 %) respondents have answered the questionnaire, of which 343 (66.6 %) were males and 172 (33.4 %) were females, ranging from 18 to 60 years [35 ± 3.4 years]. More than half of respondents (55.1 %) were 18–24 years of age, had attained the level of higher educational degree or above (63.9 %), and single (59.6 %). More than half of respondents (58.4 %) were students, and have faithfully believed in God the Almighty (78.3 %). The mean income was 25,000 ± 2000 (range 20,000–30,000) Indian Rubies-INR and mean number of adult family members living at home with their PLWHIA was 4.5 ± 1.3. The characteristics of the respondents were presented in Table 1.

**Table 1:** The respondents demographics and characteristics (n = 515)

	Number	Percentage
Gender		
Male	343	66.6
Female	172	33.4
Age		
18–24	284	55.1
25–34	122	23.7
35–44	82	15.9
45–60	27	5.2
Educational level		
Illiterate	29	5.6
Primary school	53	10.3
High school	53	10.3
Secondary	51	9.9
Higher degree	329	63.9
Marital		
Single	307	59.6
Married	186	36.1
Widow	16	3.1
Separated	6	1.2
Employer		
Student	301	58.4
Housewife	84	16.3
Employer	93	18.1
Unemployed	32	6.2
Retired	5	1
Religion		
Hindu	266	51.7
Christian	28	5.4
Muslim	39	7.6
Others	182	35.3
Belief		
Yes	403	78.3
No	68	13.2
Unsure	44	8.5
Family		
1–3	177	34.4
4–5	232	45
>equal 6	106	20.6
Income		
<20,000	199	38.6
<30,000	283	55
>30,000	33	6.4

### Respondents’ knowledge towards HIV/AIDS

#### Total scores of KAP-MV

The total mean scores of the study sample on respondent’s knowledge, attitude, perception, views and misconception (KAP-MV score) was found to be 64.9 ± 9.1. Which was categorized good (reference ranging from 56 to 82). Table 2 shows adult family members knowledge towards basic information about HIV/AIDS. The knowledge score can range between 0 and 10 and the mean knowledge score was found to be 8.0 ± 1.7. The vast majority of respondents have had right knowledge about the abbreviation of AIDS (61.4 %), AIDS is a transmittable disease (94.2 %), AIDS is a hereditary disease (87.2 %), AIDS is a curable disease (88.2 %) and that there is a vaccine for AIDS (92.2 %).

**Table 2:** Respondents knowledge and attitude about HIV/AIDS in Warangal, South India (n = 515)

	Variables	Correct answer	Correct answers (%)	Wrong answers (%)	Mean ± standard deviation
	*General knowledge*				
1.	AIDS abbreviation	Acquired Immunodeficiency Virus	316 (61.4)^a^	199 (38.6)	8.01 ± 1.76
2.	AIDS a transmittable disease	Yes	485 (94.2)	30 (5.8)	
3.	AIDS a hereditary disease	No	449 (87.2)	66 (12.8)	
4.	AIDS cured at this moment	No	454 (88.2)	61 (11.8)	
5.	There is a vaccine for AIDS	No	475 (92.2)	40 (7.8)	
	*Attitudes*				
6.	Feel comfortable talking with AIDS patients	Yes	277 (53.8)	238 (46.2)	5.80 ± 3.47
7.	Feel comfortable working with AIDS patients	Yes	275 (53.4)	240 (46.6)	
8.	Living with AIDS patients in same house	Yes	266 (51.7)^a^	249 (48.3)	
9.	Feel empathy towards AIDS patients	Yes	282 (54.8)	233 (45.2)	
10.	AIDS patients deserve free treatment	Yes	394 (76.5)	121 (23.5)	
	*Perception*				
11.	Sexual intercourse without a condom with HIV-infected person	Yes	469 (91.1)	46 (8.9)	23.44 ± 4.19
12.	Sharing needle with HIV-infected organ	Yes	499 (96.9)	16 (3.1)	
13.	Transfusion of HIV-infected blood or receiving HIV-infected organ	Yes	498 (96.7)	17 (3.3)	
14.	Having sex with multiple sexual partners with unknown HIV status	Yes	484 (94.0)	31 (6.0)	
15.	From an HIV positive mother to her fetus	Yes	479 (93.0)	36 (7.0)	
16.	Sharing personal items such as shaving blades	Yes	438 (85.0)	77 (15.0)	
17.	Breast Feeding from a HIV-infected mother	Yes	410 (79.6)	105 (20.4)	
18.	Having tattoo or body piercing	No	337 (65.4)	178 (34.6)	
19.	Kissing can transmit HIV-infection	No	147 (28.5)^a^	368 (71.5)	
20.	Mosquito bites	No	460 (89.3)	55 (10.7)	
21.	Sharing/eating a meal with an HIV-infected person	No	452 (87.8)	63 (12.2)	
22.	Sharing water or drinks with an HIV-infected person	No	465 (90.3)	50 (9.7)	
23.	Using Public toilets	No	470 (91.3)	45 (8.7)	
24.	Casual contacts (hugging or touching) with an HIV-infected person	No	427 (82.9)^a^	88 (17.1)	

^a^Most wrongly answered

#### Attitudes

The adult family members’ feeling towards PLWHA was presented in Table 2. The attitude score can range between 0 and 10 and the mean attitude score was found to be 5.8 ± 3.4. Slightly above half of respondents feel comfortable talking with their PLWHA. Slightly less than this percent feel comfortable working with PLWHA. Slightly more than half 266 (51.7 %) of the respondents, indicated that they would be able to live in the same house with a person having HIV/AIDS. However, 282 (54.8 %) of the respondents declared that they feel empathy towards PLWHA. A higher than two-third of respondents indicated that PLWHA deserve free treatment, [Table 2].

#### Perception towards possible causes of HIV infection

The sum of perception scores can range between 0 and 28 and respondents overall mean score in this domain was 23.4 ± 4.1. The analyses of data have shown that in most statements concerning perception and beliefs about the possible causes of HIV infections, the majority of respondents had good positive perception regarding HIV causes. However, some questions showed less perception towards causes of HIV/AIDS transmission. For example, only 337 (65.4 %) and 147 (28.5 %) knew that kissing and tattoos cannot transmit HIV-infections, respectively, [Table 2].

#### Respondent’s views about measures to prevent HIV infection

Overall respondent views towards preventing HIV transmission mean score was 21.0 ± 3.9 (reference range from 0 to 24). Generally, the views of adult family members were highly regarded as good towards certain issues concerning PLWHA and HIV/AIDS as a transmitted disease. For instance when respondents were asked that they avoid taking illicit drugs/use of intravenous drugs, avoiding sharing needles and syringes, having sex with only one faithful uninfected partner, using condoms during sexual intercourse and treating sexually transmitted diseases (STDs) promptly with more than 90 % had have answered correctly. On the other hand less than 90 % but more than 80 % responded correctly to the followings: screening donated blood before transfusion, not sharing food with an infected person, do not stay with infected person on same house and do not have casual contact with infected person. Furthermore, less than 80 % and over 70 % of respondents have shown correct answers to preventive measures as not sharing toilets with an infected person, isolating people living with HIV/AIDS (negatively worded) and avoid mosquito bites for HIV transmission, [Table 2].

#### Respondent’s misconception about HIV/AIDS

The majority of the respondents had less misconception about HIV/AIDS, with 65–90 % correctly answering the five statements. However, many misconceptions were still noted relating to HIV/AIDS, with 34.6 % of respondents believing that love could be a reason for HIV/AIDS and 27.6 % believing that AIDS is a punishment from the God, [Table 2].

#### Correlation analysis for outcome variables

The results shown in Table 3 demonstrated the Spearman’s (r) rank order correlation (rho) and the associations of variables with each other. Mean knowledge score was significantly associated with level of education (*p* < 0.001) and religion (*p* < 0.001). Knowledge was significantly correlated with attitude (r = −0.15, *p* < 0.001), views (0.381; *p* < 0.001), perceptions (0.39; *p* < 0.001) and total KAP-MV (0.43; *p* < 0.001) towards HIV/AIDS. Adult family members of PLWHA with less knowledge score had more negative attitude, perception and views towards preventing HIV infections. Furthermore, misconceptions were highly associated with PLWHA families with negative views (0.337; *p* < 0.001) and less perceptions (0.201; *p* < 0.001). Age, level of education, marital status, religion status and religious beliefs were significantly associated with most attitude scores, (*p* < 0.001) and were minimally associated with respondents perception about HIV/AIDS, (*p* < 0.001). Results have revealed that all the sociodemographic variables were significantly associated with respondents’ views, (*p* < 0.001). Religious status (*p* < 0.001) was significantly associated with respondents’ misconceptions. The results of the correlations were illustrated in Table 3.

**Table 3:** Respondents views and misconceptions about HIV/AIDS (n = 515)

	Variables	Correct answer	Correct answers (%)	Wrong answers (%)	Mean ± standard deviation
	*Respondents views*				
25.	Avoid taking illicit drugs/use of intravenous drugs	Yes	477 (92.6)	38 (7.4)	21.00 ± 3.98
26.	By avoiding sharing needles and syringes	Yes	501 (97.3)	14 (2.7)	
27.	Having sex with only one faithful, uninfected partner	Yes	494 (95.9)	21 (4.1)	
28.	Using condoms during sexual intercourse	Yes	491 (95.3)	24 (4.7)	
29.	Treating STDs promptly	Yes	480 (93.2)	35 (6.8)	
30.	Screening donated blood before transfusion	Yes	463 (89.9)	52 (10.1)	
31.	Not sharing toilets with an infected person	No	411 (79.8)	104 (20.2)	
32.	Not sharing food with an infected person	No	431 (83.7)	84 (16.3)	
33.	Isolating people living with HIV/AIDS	No	367 (71.3)^a^	149 (28.7)	
34.	Do not stay with infected person on same house	No	448 (87.0)	67 (13.0)	
35.	Do not have casual contact with infected person	No	439 (85.2)	76 (14.8)	
36.	Avoid mosquito bites for HIV transmission	No	406 (78.8)	109 (21.2)	
	*Misconceptions*				
37.	Love is a reason for HIV/AIDS	No	337 (65.4)^a^	178 (34.6)	8.03 ± 2.19
38.	AIDS is a punishment of God	No	373 (72.4)	142 (27.6)	
39.	AIDS can treat by holy water	No	442 (85.8)	73 (14.2)	
40.	AIDS do not come after marriage	No	454 (88.2)	61 (11.8)	
41.	AIDS can be transmitted by the cough	No	461 (89.5)	54 (10.5)	

^a^Most wrongly answered

## Discussions

The main finding of the current study was the satisfactory outcome of the KAP-MV survey in term of evaluating the adult family member basic knowledge, attitude, perception, misconception and views of their beloved PLWHA. We have used a multiple component questionnaire with diverse domains to the level of understanding of adult family members towards HIV/AIDS, feelings, perception about possible causes of HIV/AIDS, misconception towards certain myth about PLWHA and views on certain measures to prevent HIV transmissions. The instrument used in our study demonstrated clarity to communicate the intended meaning, coherency to define the logic wording, extendibility to accommodate newly added items with the domains, unbiased to allow for applicability and complex to meet the desired study purposes (Table 4).

**Table 4:** Spearman’s rho correlations analysis

Pearson correlations with outcome variables						
	Knowledge	Attitude	Respondent views	Perception	Misconceptions	Total
Knowledge	–	−0.159^**^ (0.000)	0.381^b^ (0.000)	0.395^b^ (0.000)	0.074^a^ (0.093)	0.438^b^ (0.000)
Attitude	−0.159^b^ (0.000)	–	−0.033 (0.459)	−0.078 (0.77)	0.052 (0.240)	0.385^b^ (0.000)
Respondents views	0.381^b^ (0.000)	−0.033 (0.607)	–	0.337^b^ (0.000)	0.346^b^ (0.000)	0.715^b^ (0.000)
Perception	0.395^b^ (0.000)	−0.078 (0.129)	0.337^b^ (0.000)	–	0.201^b^ (0.000)	0.624^b^ (0.000)
Misconceptions	0.074^a^ (0.016)	0.052 (0.569)	0.346^b^ (0.000)	0.201^b^ (0.000)	–	0.518^b^ (0.000)
Total	0.438^b^ (0.000)	0.385^b^ (0.000)	0.715^b^ (0.000)	0.624^b^ (0.000)	0.518^b ^(0.000)	–

^a^Correlation is significant at the 0.05 level (2-tailed)

^b^Correlation is significant at the 0.01 level (2-tailed)

Many studies has used the WHO-KABP scale to assess the knowledge, attitude and practice towards PLWHA from past decades and applied on different groups, subgroups and geographical populations. To the best of our knowledge, this is the first study focused on PLWHA adult family members KAP-MV residing in high prevalent Warangal district, South-Central India.

### Discussions on KAP-MV

#### Knowledge

Nearly two-third of respondents have had a clear knowledge about the abbreviation used for HIV/AIDS. The adult family members of PLWHA had expressed a positive answer towards HIV/AIDS and high level of KAP-MV score on issues related to HIV/AIDS transmission, considering the majority of the respondents had answered correctly. Also the notation that AIDS is a hereditary disease or AIDS is a cured disease and that there is a vaccine for AIDS were equally answered correctly by respondents in a very high percent. However, a low percent of the respondents believed that AIDS is a hereditary disease and that there is an active treatment to cure AIDS, which was consistently very low compared with other results conducted in various populations, (Meera et al. 2013; Tebourski and Ben Alaya 2004; Ayranchi 2005; Montazeri 2005). In our study, considerable proportion (7.8 %) of the respondents thought that there is vaccine for AIDS, which was lower than findings of other study (11 %), (Yazdi et al. 2006).

In overall knowledge domain, the respondents have shown a high level of knowledge towards HIV/AIDS. These findings are much better than the previous studies under taken in Behaviors Surveillance Survey (BSS) (Claeson and Alexander 2008) and in Singh and co-workers study. (Kumar et al. 2015)

### Attitudes

With the exception of the statement that AIDS patients deserve free treatment, the respondents demonstrated a fair attitude associated with 4 of the 5 items in this domain. It was found that some appreciated number of adult family members with negative attitude have expressed less tolerance in talking, working, willing to live and feeling empathy towards PLWHA.

A report from the Japan demonstrated a positive change in attitude among college students, (Wang et al. 2013). Other (Lal et al. 2008) study performed on demonstrated a positive association between positive attitude concerning AIDS and tolerance of HIV patients. We have found slightly more than half of respondents expressed more tolerated attitude towards AIDS patients. Though better knowledge had not shown their changing attitude, we have anticipated that PLWHA adult family members need necessary counseling and targeted tailored education about attitude to pose some influence upon behavioral and attitudinal changes.

### Perception

Overall, there were misperception was observed regarding the kissing can transmit HIV. This problem was also addressed by previous investigators (Yazdi et al. 2006; Fonner et al. 2014; Nkansah-Amankra et al. 2011). However, one study (Yazdi et al. 2006) shown many misconceptions regarding HIV transmission than our study population. In our study, a considerable proportion of respondents thought that sexual intercourse without condom usage can transmit HIV/AIDS. Using condom is important preventive measure to prevent HIV transmission. Other methods are avoiding unsafe sex, avoiding untested blood transfusion and sharing personal items like blades, needles and syringes. Regarding the using of sterilized needles and syringes and HIV free blood transfusion, most of the respondents answered correctly indicating the important preventive measure for HIV transmission. These results were much higher than in the study by Kumar and co-workers. (Goel et al. 2010) Study on nursing students where 75 % of the respondents agreed that using of sterilized needles and syringes, HIV free blood transfusion (82 %) were important for prevention. PLWHA adult family member perception towards preventing HIV-infections is very important in developing prevention interventions strategies.

### Views towards preventive measures

The most interesting finding from this survey was the fact that adult family members have showed positive opinion towards PLWHA, with the exception of few items (31, 33 and 36). For example, 28.7 % of the respondents opinioned that PLWHA should be isolated from the community or special centre, avoiding mosquito bites to prevent HIV transmission (21.2 %) and unwillingness to share toilets with HIV infected people. Furthermore, when taking into consideration that the majority of the respondents had a positive views towards PLWHA and the level of opinions about PLWHA were above average for this attitude may be that few of adult family members do not have much positive opinion or views towards people with AIDS.

### Misconception

Our study demonstrated that in general average number of the respondents harbored misconceptions. For instance, 34.5 % of the adult family members agreed with the statement that love is a reason for HIV/AIDS and AIDS is a punishment from God (27.6 %). In parallel, this proportion was slightly higher than in Ayranci and co-workers study (23.2 %), (Tebourski and Ben Alaya 2004) and double than findings from Montazeri study (14.2 %), (Montazeri 2005) which was conducted on Iranian general population. This means that these adult family members believe that even relationship factors and religious factors could not prevent a person from HIV infection. This indicated that there is a need for further investigation into the role of positive relationship attitude and religion in AIDS prevention, particularly in India where religion beliefs plays an important role in people’s everyday life.

### The KAP-MV correlations

The adult family members’ role is very important, to support, care and help their beloved PLWHA to improve their quality of life. Taking everything into account, overall, there was a little negative attitude, low perceptions and misconception about HIV/AIDS was observed in the responded adult family members. These may be a risk probably influenced by the widespread denial of the existence of false beliefs from the Indian communities and disparities in attitudinal problems aroused by the lack of awareness about AIDS which need to be addressed.

### Study limitations

The present study reported here has confronted several limitations. First, the study was designed as self-administered questionnaire, which poses difficulty in validating the PLWHA adult family member answers. They may over-report socially desirable answers and underreport undesirable ones.

Secondly, we believed the participants were honest related to relationship to the PLWHA, so we did not included them in the questionnaire due to ethical and cultural concern in India. However, since relationship status and caring status are considered to be the major avenues to support PLWHA patients, this omission was unfortunate. The inclusion of such data would have provided useful information on the caring and support activities among PLWHA adult family members.

Finally, these findings may not be extrapolated to other populations groups in India who may differ substantially in relationships, distribution, awareness and cultural status.

## Strength and weakness of the study

The PLWHA adult family members represent an important subgroup of the Indian population affect and affected by the provided care. The information provided will be useful in the planning and focusing of future awareness programs and interventions related to HIV/AIDS in PLWHA adult family members.

## Conclusions

Adult family members of PLWHA had shown accepted satisfactory levels (64.9 %) of KAP-MV towards HIV/AIDS high prevalent in Warangal district, South India. The level of education, marital status, religious beliefs, and employment status has been identified as key barriers for adult family members caring for PLWHA. Interventions targeting adult family members of PLWHA are warranted to raise their awareness and knowledge, improves attitudes, minimize the negative views and misconception for more proactive role.

## Practice implications

The following are highlighted as implications of the study on clinical practice:

- Encourage the role of family members for supporting PLWHA.
- Deploy interventions to raise the knowledge, attitudes, perception and views of family members of PLWHA.
- Minimize the barriers for adult family members caring for PLWHA.
- Change the family members misconceptions about PLWHA.

## Abbreviations

AIDS: acquired immune deficiency syndrome
ART: antiretroviral therapy
BSS: behaviors surveillance survey
HIV: human immune virus
KAP-MV: knowledge, attitudes, perception, misconception and views
KABP: knowledge, attitudes, beliefs and practices
MGM: Mahatma Gandhi Memorial
NACO: National Control Organization
NFHS-3: National Family Health Survey-3
NRTIs: nucleoside analogue reverse transcriptase inhibitors
NNRTIs: non nucleoside analogue reverse transcriptase inhibitors
PLWHA: people living with human immune virus-AIDS
STDs: sexually transmitted diseases
WHO: World Health Organization
